# Factors associated with medical students’ self-regulated learning and its relationship with clinical performance: a cross-sectional study

**DOI:** 10.1186/s12909-022-03186-0

**Published:** 2022-02-25

**Authors:** Jia-Yu Zhang, Yi-Juan Liu, Tao Shu, Ming Xiang, Zhan-Chun Feng

**Affiliations:** 1 School of Medicine and Health Mangement, Tongji Medical College, Huazhong University of Science and Technology, No. 13 Hangkong Road, Qiaokou Region, Wuhan City, Hubei Province China; 2 Medical academic affairs office, Tongji Medical College, Huazhong University of Science and Technology, Wuhan, China

**Keywords:** Self-regulated learning, Clinical skill, Clinical performance, Medical education

## Abstract

**Background:**

The importance of self-regulated learning (SRL) has been broadly recognised by medical education institutions and medical professionals. Self-regulated learning, which is a context-specific process, is affected by personal, contextual and social factors. Although many studies on exploring the factors that influenced SRL and the relationship of between SRL and clinical achievement levels have been carried out in western countries, little is known about the factors associated with self-regulated learning and its relationship with clinical performance among medical students in China.

**Methods:**

A cross-sectional online survey was distributed to 3rd year clinical medicine students who were in the clinical clerkship stage in a medical college in Wuhan. We used Self-regulated Learning Scale for Undergraduates (SLSU) to measure the self-regulated learning of students and Objective Structured Clinical Examination (OSCE) in the national proficiency test to assess the clinical performance of students. The participation rate was 73.95% (193 students). An independent t-test and analysis of variance were used to analyse the factors associated with self-regulated learning. The relationship between self-regulated learning and clinical performance was analysed with multilinear regression analysis.

**Results:**

Univariate analysis showed that having a clear career planning and a professional idol, providing full-time teaching clinical teachers in the clerkship department and seeking the help of the surrounding classmates and the guidance of teachers or senior students were significant predictors of self-regulated learning. Multilinear regression analysis has revealed a positive relationship among extrinsic goals (partial *r* = 0.171), clinical clerkship evaluation (partial *r* = 0.197) and clinical performance (F = 4.070, *p* = 0.004).

**Conclusions:**

Motivation-related personal and social factors related to clinical context could promote the SRL level of medical students in China. Extrinsic goals and clinical clerkship evaluation could facilitate students’ clinical achievements on clinical skills. External support, such as clinical clerkship management, might improve clinical performance on clinical skills in clinical clerkship context.

**Supplementary Information:**

The online version contains supplementary material available at 10.1186/s12909-022-03186-0.

## Background

Self-regulated learning is the process of being metacognitive, motivational and behavioural proactive in the learning process according to the theoretical model of Zimmerman [1]. This model consists of three phases: forethought, performance and self-reflection phases. More specifically, self-regulated learners effectively set goals; plan and use strategies to achieve their goals; manage their resources in the forethought phase; seek to manage emotions, cognition and behaviour to attain their forethought goals in the performance phase; self-evaluate whether they have attained their goals and modify or adapt their goals or strategies for future learning in the self-reflection phase [2, 3].

Self-regulation is a complex interactive process of physical and mental activities, which are affected by personal, social and contextual attributes [2, 4, 5]. Personal and social attributes are closely related to its three-phases process [6]. Studies have shown that goals have a motivational function on SRL [7, 8]. The use of goal setting and learning plans allow for adaptation to person and context in the forethought phases. Personal attributes found to affect SRL on a more general level of students in China included grade and major [9, 10]. In addition to these factors, studies showed that financial status and clinical internship experience could affect SRL in medical students, to name a few [11, 12]. The support of a coach or mentor might be a prerequisite for the SRL process to develop to its full potential in the performance phases. Social attributes affecting SRL of paediatric residents in North American countries pertained to the influence of supervisor and peers [13], faculty staff support [14], among others. Contextual attributes known to affect self-regulation of medical students in a classroom setting in European or North American countries included available facilities and curriculum pedagogy, among others [15–17].

One of the important factors contributing to enhance students’ academic performance was self-regulated learning. Self-regulated students were more effective learners who attained higher grades during high school and in non-medical college [18–20]. Besides, many studies have adopted different research methods to demonstrate this positive relationship in medical students [21–23]. Students who were successful in venipuncture had strong high levels of strategic thinking using SRL microanalysis, which was a tool to examine the self-regulatory profiles of successful performers and strugglers across three phases [3]. In addition, positive relations of monitoring, reflection and effort with first-year GPA of students were found using the Self-Regulation of Learning Self-Report Scale [23]. Furthermore, correlation analysis showed significant association of autonomous motivation with higher levels of academic achievement using the Academic Motivation Scale [21]. OSCE scores of the students were observed to increase in conjunction with increased self-efficacy levels using Motivated Strategies for Learning Questionnaire [22].

Self-regulated learning is an important strategy for the continuous professional development of medical students [24]. Many studies on exploring the factors influencing SRL and the relationship of SRL and achievement levels have been carried out. However, to our knowledge, research on SRL of medical students in China during clinical clerkship is largely lacking. Moreover, no research has explored the relationship between SRL and clinical performance in Chinese medical students. Considering the huge difference between clinical medical education in western countries and China, carrying out research on SRL was necessary in the domestic clinical context. Besides, family economy and family culture, which might be potential confounding factors, could improve students’ academic performance according to the family capital theory. Therefore, we included family economy variables such as area of residence and household income and family culture variables such as parents’ educational level and parents’ expectations into this study [25]. This study focused on the factors associated with self-regulated learning and its relationship with clinical skills during their clinical clerkship. The following questions were put forth:

1. What factors affect medical students’ self-regulated learning in their clinical clerkship?
2. Is there a relationship between the self-regulated learning ability of medical students and clinical performance in the clinical clerkship?

## Method

### Setting

We conducted a cross-sectional survey among students enrolled in different clerkships at the beginning of the rotational stage. The target population consisted of medical students majoring in Clinical Medicine (5-year schooling) at the Tongji Medical College Huazhong University of Science and Technology, China. The clinical medicine project, which adopts traditional and PBL teaching methods, includes a pre-clinical phase (Years 1–2.5) and a clinical phase (Years 2.5–5). The clinical phase consists of rotational clerkships ranging from 2 weeks to 16 weeks in duration. The electronic survey, which was anonymous and confidential, was distributed electronically by WeChat tools to 261 students in the clinical clerkship stage on June 2021 with the Wenjuanxing platform.

### Data collection

The study participants were 3rd year medical students, and 193 students completed the questionnaire (73.95% response rate). Among these 193 students, 150 (77.72%) have correctly filled in their student ID. Therefore, we could obtain their clinical skill scores and age according to their student ID. To maximise the use of data, all analyses except samples with incorrect student IDs when it came to the analysis of clinical skill scores, were regarded as valid samples. The male/female ratio was approximately 1:1 (Male: 97, Female: 96) and the mean age was 22.3 ± 0.88. The study was approved by the institutional review boards of the Tongji Medical College Huazhong University of Science and Technology.

### Instruments

#### Self-regulated learning scale for undergraduates

SLSU was developed by Chinese researchers on the basis of Zimmerman’s self-regulated learning theory according to the actual situation of college students in China [26]. This scale was divided into two subscales: the learning motivation scale (LMS) and the learning strategy scale (LSS). Items were scored on a 5-point Likert scale ranging from one (totally disagree) to five (totally agree). Additionally, the expressions of some items were modified according to the characteristics of clinical clerkship (e.g. changing the following ‘I often found pleasure in the study’ to ‘I often found pleasure in clinical clerkship’). A total of 7 items (e.g. ‘I arrange the time to do the most important thing first’) were deleted because of its inapplicability in the clinical setting.

LMS was made up of 25 items covering five domains of learning motivation: self-efficacy (six items), intrinsic goals (eight items), control beliefs about learning (seven items), extrinsic goals (two items) and clinical clerkship value (two items). LSS was made up of 37 items covering three domains of learning strategy: test anxiety (four items), general method (five items), help seeking during clinical clerkship (eight items), study plan during clinical clerkship (six items), clinical clerkship summary (five items), clinical clerkship evaluation (three items) and clinical clerkship management (four items). The reliability of the subdimensions for the motivation dimension ranged between 0.74 and 0.97, whilst this value ranged between 0.74 and 0.93 for the learning strategies dimension (Table 1).

**Table 1:** The mean of SLSU scores of students and the Cronbacha coefficient of SLSU

Scale	Items	Mean	SD	Alpha
Self-regulated learning		3.53	0.60	0.98
Learning motivation		3.53	0.64	0.97
	Self-efficacy	3.39	0.83	0.92
	Intrinsic goals	3.78	0.81	0.95
	Control beliefs about learning	3.65	0.72	0.92
	Extrinsic goals	3.05	0.95	0.74
	Clinical clerkship value	3.76	0.92	0.94
Learning strategy		3.52	0.60	0.96
	Test anxiety	3.58	0.84	0.91
	General method	3.70	0.73	0.92
	Help seeking during clinical clerkship	3.49	0.75	0.93
	Study plan during clinical clerkship	3.42	0.80	0.93
	Clinical clerkship summary	3.36	0.81	0.93
	Clinical clerkship evaluation	3.50	0.80	0.85
	Clinical clerkship management	3.64	0.80	0.74

#### Clinical performance

The clinical skill test, which was evaluated by the Objective Structured Clinical Examination in the national proficiency test, was used to assess clinical performance of the students. The OSCE includes 6 stations included in taking patient history (Stations I and II), physical examination skills (Stations III and IV) and basic clinical skills (Stations V and VI).

### Statistical analysis

Statistical analyses were performed with SPSS for Windows version 21 and the statistical computing language R [27]. Categorical variables were presented as numbers and percentages and continuous variables as mean ± standard deviation (SD). Continuous variables with Student’s t-tests or analysis of variance. Multivariate linear regression analysis was conducted to evaluate the association between self-regulated learning and clinical performance. The assumptions of the multilinear regression analysis were investigated before performing the analysis. Variance inflation factor (VIF) was used to examine the multicollinearity of the regression analysis. Durbin-Watson (DW) autocorrelation statistic was generated to identify models with serial autocorrelation. A value of VIF higher than 10 was considered to be a multicollinearity, and a value of DW between 1.5 and 2.5 was considered to have no autocorrelation [28]. A *p*-value of 0.05 (two-tailed) was considered to be statistically significant. In this study, the values of VIF (1.248–7.226) and DW (1.837) were both in the allowable range.

## Results

### Descriptive statistics of personal characteristics and environmental factors

Out of the 261 students who were invited to participate, 193 responses were received giving an overall response rate of 73.95%. The mean scores ranged between 3.05 and 3.78 in the LMS section and between 3.36 and 3.64 in the LSS section. The overall mean score and reliability coefficient of the SLSU were 3.49 and 0.98, respectively (Table 1). The numbers (percentages) of municipalities or provincial capitals, prefecture-level cities, county-level cities and town or rural areas were 39 (20.2%), 56 (29%), 53 (27.5%) and 45 (23.3%), respectively. Over four-fifths (84.4%) of the students’ family income were below 200,000 (CNY). Approximately 90 respondents had a professional idol and a clear career planning. A total of 77 students have encountered a full-time teaching clinical teacher in the current clerkship department. Approximately 170 students sought the help of their surrounding classmates and the guidance of teachers or senior students. Additionally, 112 students experienced medical disputes during their clinical clerkship (Additional file 1).

### Analysis of differences in SRL according to personal characteristics and environmental factors

Table 2 displays the differences in the means and standard deviations of self-regulated learning and its subscales among individual and environmental feature groups. Among these factors, a professional idol (*p* = 0.029), a clear career planning (*p* = 0.001), full-time clinical teaching teachers (*p* = 0 .041), the help of surrounding classmates (*p* < 0.001) and the guidance of teachers or senior students (*p* < 0.001) had a univariate correlation with the self-regulated learning ability. However, no differences existed in personal characteristics, such as gender, area of residence, among others. This study also analysed the relationships between these variables and learning motivation and learning strategy. Table 2 displays the detailed statistical results.

**Table 2:** Differences in self-regulated learning and its subscales by personal characteristics and environmental factors

Variables	Items	Learning motivation		Learning strategy		Self-regulated learning	
		Mean ± SD	*p*	Mean ± SD	*p*	Mean ± SD	*p*
Gender	Male	3.50 ± 0.73	0.420	3.53 ± 0.67	0.711	3.52 ± 0.67	0.805
	Female	3.57 ± 0.54		3.50 ± 0.53		3.54 ± 0.52	
Area of residence	Municipalities/Provincial capitals	3.54 ± 0.63	0.514	3.53 ± 0.58	0.532	3.53 ± 0.58	0.490
	Prefecture-level city	3.63 ± 0.67		3.59 ± 0.63		3.61 ± 0.63	
	County-level cities	3.44 ± 0.73		3.42 ± 0.64		3.43 ± 0.66	
	Town/Rural areas	3.52 ± 0.50		3.52 ± 0.53		3.52 ± 0.49	
Parents’ highest education level	Elementary School	3.45 ± 1.02	0.863	3.37 ± 0.8	0.721	3.41 ± 0.9	0.810
	Junior High School	3.54 ± 0.65		3.52 ± 0.62		3.53 ± 0.61	
	Senior High School	3.45 ± 0.37		3.44 ± 0.40		3.45 ± 0.37	
	Technical Secondary School/Junior College	3.53 ± 0.74		3.61 ± 0.69		3.57 ± 0.69	
	Undergraduate	3.54 ± 0.60		3.48 ± 0.6		3.51 ± 0.56	
	Master	3.75 ± 0.35		3.67 ± 0.31		3.71 ± 0.33	
	Doctor	3.84 ± 0.29		3.79 ± 0.24		3.81 ± 0.24	
Parents’ expectations of your education	Undergraduate	3.46 ± 1.01	0.769	3.46 ± 0.8	0.420	3.46 ± 0.89	0.586
	Master	3.50 ± 0.50		3.45 ± 0.49		3.48 ± 0.48	
	Doctor	3.56 ± 0.67		3.57 ± 0.63		3.56 ± 0.62	
Average annual household income (CNY)	50,000 and bellow	3.44 ± 0.76	0.629	3.44 ± 0.68	0.461	3.44 ± 0.7	0.529
	50,000–100,000	3.52 ± 0.63		3.47 ± 0.55		3.49 ± 0.57	
	100,000–200,000	3.62 ± 0.56		3.59 ± 0.63		3.61 ± 0.56	
	200,000–400,000	3.62 ± 0.50		3.68 ± 0.53		3.65 ± 0.48	
	400,000 and above	3.50 ± 0.77		3.50 ± 0.48		3.5 ± 0.61	
Whether to have a professional idol	Yes	3.63 ± 0.66	0.063	3.63 ± 0.59	**0.019**	3.63 ± 0.61	**0.029**
	No	3.45 ± 0.61		3.42 ± 0.59		3.44 ± 0.58	
Whether to have a clear career planning	Yes	3.67 ± 0.71	**0.005**	3.68 ± 0.66	**0.001**	3.68 ± 0.66	**0.001**
	No	3.42 ± 0.56		3.38 ± 0.50		3.4 ± 0.51	
Whether to have full-time teaching clinical teachers in the current clerkship department	Yes	3.67 ± 0.60	**0.018**	3.61 ± 0.61	0.072	3.64 ± 0.58	**0.029**
	No	3.45 ± 0.65		3.45 ± 0.59		3.45 ± 0.6	
Whether to seek the help of the surrounding classmates	Yes	3.62 ± 0.57	**< 0.001**	3.61 ± 0.56	**< 0.001**	3.61 ± 0.54	**< 0.001**
	No	2.99 ± 0.83		2.91 ± 0.50		2.95 ± 0.64	
Whether to seek the guidance of teachers or senior students	Yes	3.61 ± 0.58	**< 0.001**	3.58 ± 0.58	**< 0.001**	3.59 ± 0.55	**< 0.001**
	No	2.92 ± 0.82		2.97 ± 0.55		2.94 ± 0.67	
Whether to experience medical dispute during clinical clerkship	Yes	3.56 ± 0.65	0.514	3.54 ± 0.59	0.534	3.55 ± 0.60	0.507
	No	3.5 ± 0.63		3.49 ± 0.62		3.49 ± 0.60	

Statistically significant values are indicated in bold

### Relationship between self-regulated learning and clinical performance

We used the method of backward stepwise regression to screen variables that had an impact on the level of students’ clinical skills. The subdimensions of learning motivations and learning strategies indicated a weak but meaningful relationship with the OSCE scores (F = 4.070, *p* = 0.004; *R*^2^ = 0.098, Adjust-*R*^2^ = 0.074) (Table 3). According to the standardised regression coefficients, the most important subdimensions relative to OSCE score were clinical clerkship evaluation and extrinsic goals.

**Table 3:** The Multiple Liner Regression Analysis Results for Students’ Objective Structured Clinical Exam Score

Items	B	SE	b	t	*p*	95%CI		Partial r
						lower	upper	
Constant	73.046	3.393		21.528	< 0.001	66.342	79.751	
Control beliefs about learning	−1.998	1.015	−0.221	−1.968	0.051	−4.005	0.008	−0.159
Extrinsic goals	1.223	0.575	0.191	2.125	**0.035**	0.086	2.360	0.171
Clinical clerkship evaluation	2.223	0.904	0.280	2.46	**0.015**	0.437	4.009	0.197
Clinical clerkship management	1.159	0.612	0.156	1.893	0.060	−0.051	2.369	0.153

Statistically significant values are indicated in bold, F = 4.070, *p* = 0.004; *R*^2^ = 0.098, Adjust-*R*^2^ = 0.074

Clinical clerkship evaluation and extrinsic goals were positively correlated (*r* = 0.197, *r* = 0.171) with OSCE scores at the statistical level of 0.05. Clinical clerkship management was positively correlated (*r* = 0.153) with OSCE scores, whilst control over learning beliefs were negatively correlated (*r* = − 0.159) with OSCE scores at the statistical level of 0.1.

## Discussion

By exploring factors that influenced medical students’ self-regulated learning in clinical setting, our study revealed that motivation-related personal factors and social factors related to clinical context could promote the SRL level of students. Learning motivation (extrinsic goals) and learning strategy (clinical clerkship evaluation) were positively associated with students’ clinical achievements on clinical skills. External support, such as clinical clerkship management, might improve clinical skills in clinical setting.

### What factors influence the self-regulated learning levels of medical students during clinical clerkship?

Most of the factors associated with self-regulated competence were motivation-related factors. Students with a clear career plan and have a professional idol had a higher level of SRL. This result supported the arguments of Zimmerman theory, which suggested that self-motivation beliefs such as self-efficacy and goal orientation provide the impetus or motivation for a learner to put forth the necessary effort to engage in the self-regulation process [29]. A clear career plan and with professional idols might improve students’ self-efficacy, thereby enhancing their independent learning in the process of clinical practice. This finding was in line with previous reports of SRL in other settings, which stated that goals could function as regulatory agents for SRL [8].

Earlier research on SRL in classroom settings have proven that direct instruction by teachers or opportunities for modelling these strategies by senior students could support SRL [30, 31]. Our research results, conducted in the clinical settings, were consistent with those found in the classroom settings. Social-cognitive models of self-regulation suggest that individuals are active participants who seek control over important events through the regulation of their thoughts and actions to achieve personal goals in the performance phase [32]. The possible reason could be that peer-assisted learning was considered to ease the learning process and mobilise students’ initiative in learning [33].

Although many medical undergraduate learners naturally exhibit adaptive self-regulation processes, one or more of these processes may be under developed in any given student, and most students do not fully develop the competency they require to adequately self-regulate their learning independently [3, 7]. Students who have experienced a clerkship in a department with a full-time instructor had strong SRL ability. Full-time tutors could devote extra time and energy to guide students. They might also be more familiar with the strategy of SRL. Therefore, they were conducive to the improvement of students’ SRL ability, especially for those students with a large number of deficiencies.

### Is there a relationship between medical students’ self-regulated learning competence and clinical skills in the clinical clerkship?

The relation between self-regulated learning skills and academic performance is limited [23]. The theoretical model of Zimmerman regarded goals as a critical element in the self-regulation process. Intrinsic goal orientation for learning was correlated with the higher levels of performance conducted in the classroom setting [34–36]. We found that extrinsic goals were correlated with the higher levels of OSCE in clinical clerkship. In addition, the results showed that clinical management had an impact on clinical performance. These findings might be caused by the time conflict between clinical clerkship and postgraduate entrance examinations in the domestic medical education system. A majority of students were more likely to prepare for the examination to obtain better career development prospects during their clerkship stage. This scenario led to a lower sense of self-efficacy for clinical clerkship compared with postgraduate entrance examinations in medical students. Although self-efficacy was a key component in the learning forethought process according to Zimmerman theory, the external support such as clinical clerkship management and the extrinsic goals such as grades ranking might be important for learning clinical skills in China [1].

Results showed that a weak negative correlation exists between control beliefs about learning and OSCE score but did not reach statistical significance. Interestingly, this phenomenon was also found in another study that reported the partial r of − 0.17 conducted in surgical clerkship in Turkey [22]. The attainment of clinical skills is difficult to accomplish by one person and must therefore be done in cooperation. Students need support such as an experienced tutor to improve their clinical skills in the clinical environment. The conclusion that additional external support was needed in learning the clinical skills during clinical clerkship was also found in the analysis of learning strategy. Learning strategy such as clinical clerkship evaluation could promote the improvement of clinical skills. Greater use of learning strategies such as elaboration and critical thinking were associated with higher levels of performance in a medical gross anatomy course [34]. Students could rely on elaboration and critical thinking to improve academic performance in theoretical courses. However, additional external support, especially in China, might be needed to improve clinical skills in the clinical practice process.

### Limitations

The study currently contains some limitations. First is the response bias due to the small sample size, which might have caused the sampling to miss the students who are poor at self-regulation and whose motivation to respond to the online questionnaire was low. Secondly, no standardised scale exists for measuring students’ self-regulated learning ability. The scale which was widely used in China for investigation might compensate for the obstacles compared with peers worldwide. Moreover, owing to the single institution design of the study, care must be taken not to overinterpret our findings, particularly with respect to transitions in other medical schools and countries. Furthermore, although personal characteristics such as age were not gathered in the survey, age were obtained according to their student ID and had no impact on clinical performance. In the end, the unbalanced personal characteristics (e.g. whether to seek the help of the surrounding classmates and whether to seek the guidance of teachers or senior students) data and the unknown prior achievement level may adversely affect the results.

## Conclusion

The findings obtained from this study showed that motivation-related personal factors (clear career planning and professional idol) and social factors (full-time teaching clinical teachers, the help of the surrounding classmates and the guidance of teachers or senior students) were significant predictors of self-regulated learning. Additionally, a positive relationship exists between extrinsic goals and clinical clerkship evaluation and clinical performance. Further research is also needed to identify the effect of external support on clinical academic performance in clinical setting. Considering that self-regulated learning in the clinical environment is a complex process that results from an interaction between person and context, further investigation on risk factors is necessary in the clinical environment of self-regulated learning.

## Supplementary Information

**Additional file 1.**

## Data Availability

The datasets used and/or analysed during the current study are in Chinese and are available from the corresponding author on reasonable request but will require translation to English.

## Abbreviations

SRL: Self-regulated Learning
SLSU: Self-regulated Learning Scale for Undergraduates
OSCE: Objective Structured Clinical Examination
LMS: Learning Motivation Scale
LSS: Learning Strategy Scale
SD: Standard deviation
VIF: Variance inflation factor
DW: Durbin-Watson
MSLQ: Motivated Strategies for Learning Questionnaire
