# Supplementary material for: Factors associated with medical students’ self-regulated learning and its relationship with clinical performance: a cross-sectional study
Source: BMC Med Educ. 2022 Feb 25;22:128. doi: 10.1186/s12909-022-03186-0 (PMC8876085; doi:10.1186/s12909-022-03186-0)
Supplement: Supplementary file 1 — Additional file 1. [file 12909_2022_3186_MOESM1_ESM.docx]

File1 Descriptive analysis of personal characteristics

| **Variables** | **Items** | **N(%)** |
| --- | --- | --- |
| Gender | Male | 97(50.3) |
|  | Female | 96(49.7) |
| Area of residence | Municipalities/provincial capitals | 39(20.2) |
|  | Prefecture-level city | 56(29) |
|  | County-level cities | 53(27.5) |
|  | Town/Rural areas | 45(23.3) |
| Parents' highest education level | Elementary School | 14(7.3) |
|  | Junior High School | 43(22.3) |
|  | Senior High School | 27(14) |
|  | Technical Secondary School/Junior College | 42(21.8) |
|  | Undergraduate | 54(28) |
|  | Master | 9(4.7) |
|  | Doctor | 4(2.1) |
| Parents' expectations of your education | Undergraduate | 14(7.3) |
|  | Master | 113(58.5) |
|  | Doctor | 66(34.2) |
| Average annual household income (CNY) | 50,000 and bellow | 50(25.9) |
|  | 50,000–100,000 | 61(31.6) |
|  | 100,000–200,000 | 52(26.9) |
|  | 200,000–400,000 | 20(10.4) |
|  | 400,000 and above | 10(5.2) |
| Whether to have a professional idol | Yes | 90(46.6) |
|  | No | 103(53.4) |
| Whether to have a clear career planning | Yes | 89(46.1) |
|  | No | 104(53.9) |
| Whether to have full-time teaching clinical teachers in the current clerkship department | Yes | 77(39.9) |
|  | No | 116(60.1) |
| Whether to seek the help of the surrounding classmates | Yes | 168(87) |
|  | No | 25(13) |
| Whether to seek the guidance of teachers or senior students | Yes | 173(89.6) |
|  | No | 20(10.4) |
| Whether to experience medical dispute during clinical clerkship | Yes | 112(58) |
|  | No | 81(42) |

File 2. Differences in self-regulated learning and its subscales by Demographic characteristics and learning-related characteristics.

| Variables | Items | Learning motivation | | Learning strategy | | Self-regulated learning | |
| --- | --- | --- | --- | --- | --- | --- | --- |
|  |  | Mean ± SD | p | Mean ± SD | p | Mean ± SD | p |
| Gender | Male | 3.50±0.73 | 0.420 | 3.53±0.67 | 0.711 | 3.52±0.67 | 0.805 |
|  | Female | 3.57±0.54 |  | 3.50±0.53 |  | 3.54±0.52 |  |
| Area of residence | Municipalities/  Provincial capitals | 3.54±0.63 | 0.514 | 3.53±0.58 | 0.532 | 3.53±0.58 | 0.490 |
|  | Prefecture-level city | 3.63±0.67 |  | 3.59±0.63 |  | 3.61±0.63 |  |
|  | County-level cities | 3.44±0.73 |  | 3.42±0.64 |  | 3.43±0.66 |  |
|  | Town/Rural areas | 3.52±0.50 |  | 3.52±0.53 |  | 3.52±0.49 |  |
| Parents' highest education level | Elementary School | 3.45±1.02 | 0.863 | 3.37±0.8 | 0.721 | 3.41±0.9 | 0.810 |
|  | Junior High School | 3.54±0.65 |  | 3.52±0.62 |  | 3.53±0.61 |  |
|  | Senior High School | 3.45±0.37 |  | 3.44±0.40 |  | 3.45±0.37 |  |
|  | Technical Secondary School/Junior College | 3.53±0.74 |  | 3.61±0.69 |  | 3.57±0.69 |  |
|  | Undergraduate | 3.54±0.60 |  | 3.48±0.6 |  | 3.51±0.56 |  |
|  | Master | 3.75±0.35 |  | 3.67±0.31 |  | 3.71±0.33 |  |
|  | Doctor | 3.84±0.29 |  | 3.79±0.24 |  | 3.81±0.24 |  |
| Parents' expectations of your education | Undergraduate | 3.46±1.01 | 0.769 | 3.46±0.8 | 0.420 | 3.46±0.89 | 0.586 |
|  | Master | 3.50±0.50 |  | 3.45±0.49 |  | 3.48±0.48 |  |
|  | Doctor | 3.56±0.67 |  | 3.57±0.63 |  | 3.56±0.62 |  |
| Average annual household income(CNY) | 50,000 and bellow | 3.44±0.76 | 0.629 | 3.44±0.68 | 0.461 | 3.44±0.7 | 0.529 |
|  | 50,000–100,000 | 3.52±0.63 |  | 3.47±0.55 |  | 3.49±0.57 |  |
|  | 100,000–200,000 | 3.62±0.56 |  | 3.59±0.63 |  | 3.61±0.56 |  |
|  | 200,000–400,000 | 3.62±0.50 |  | 3.68±0.53 |  | 3.65±0.48 |  |
|  | 400,000 and above | 3.50±0.77 |  | 3.50±0.48 |  | 3.5±0.61 |  |
| Whether to have a professional idol | Yes | 3.63±0.66 | 0.063 | 3.63±0.59 | **0.019** | 3.63±0.61 | **0.029** |
|  | No | 3.45±0.61 |  | 3.42±0.59 |  | 3.44±0.58 |  |
| Whether to have a clear career planning | Yes | 3.67±0.71 | **0.005** | 3.68±0.66 | **0.001** | 3.68±0.66 | **0.001** |
|  | No | 3.42±0.56 |  | 3.38±0.50 |  | 3.4±0.51 |  |
| Whether to have full-time teaching clinical teachers in the current clerkship department | Yes | 3.67±0.60 | **0.018** | 3.61±0.61 | 0.072 | 3.64±0.58 | **0.029** |
|  | No | 3.45±0.65 |  | 3.45±0.59 |  | 3.45±0.6 |  |
| Whether to seek the help of the surrounding classmates | Yes | 3.62±0.57 | **<0.001** | 3.61±0.56 | **<0.001** | 3.61±0.54 | **<0.001** |
|  | No | 2.99±0.83 |  | 2.91±0.50 |  | 2.95±0.64 |  |
| Whether to seek the guidance of teachers or senior students | Yes | 3.61±0.58 | **<0.001** | 3.58±0.58 | **<0.001** | 3.59±0.55 | **<0.001** |
|  | No | 2.92±0.82 |  | 2.97±0.55 |  | 2.94±0.67 |  |
| Whether to experience medical dispute during clinical clerkship | Yes | 3.56±0.65 | 0.514 | 3.54±0.59 | 0.534 | 3.55±0.6 | 0.507 |
|  | No | 3.5±0.63 |  | 3.49±0.62 |  | 3.49±0.6 |  |

File 3. F/t, p, effect size of the analyses of variance and t test

| Variables | Learning motivation | | | Learning strategy | | | Self-regulated learning | | |
| --- | --- | --- | --- | --- | --- | --- | --- | --- | --- |
|  | F/t | p | Effect size | F/t | p | Effect size | F/t | p | Effect size |
| Gender | 0.808 | 0.420 | 0.109 | 0.371 | 0.711 | 0.05 | 0.248 | 0.805 | 0.033 |
| Area of residence | 0.766 | 0.514 | 0.012 | 0.736 | 0.532 | 0.012 | 0.809 | 0.490 | 0.013 |
| Parents' highest education level | 0.424 | 0.863 | 0.013 | 0.611 | 0.721 | 0.019 | 0.497 | 0.810 | 0.016 |
| Parents' expectations of your education | 0.263 | 0.769 | 0.003 | 0.871 | 0.420 | 0.009 | 0.536 | 0.586 | 0.006 |
| Average annual household income(CNY) | 0.648 | 0.629 | 0.014 | 0.907 | 0.461 | 0.019 | 0.797 | 0.529 | 0.017 |
| Whether to have a professional idol | 1.873 | 0.063 | 0.283 | **2.362** | **0.019** | **0.360** | **2.190** | **0.030** | **0.319** |
| Whether to have a clear career planning | **2.830** | **0.005** | 0.391 | **3.539** | **0.001** | **0.512** | **3.307** | **0.001** | **0.475** |
| Whether to have full-time teaching clinical teachers in the current clerkship department | **2.389** | **0.018** | **0.352** | 1.808 | 0.072 | 0.267 | **2.196** | **0.029** | **0.322** |
| Whether to seek the help of the surrounding classmates | **4.787** | **<0.001** | **0.885** | **5.913** | **<0.001** | **1.319** | **5.573** | **<0.001** | **1.115** |
| Whether to seek the guidance of teachers or senior students | **4.773** | **<0.001** | **0.972** | **4.531** | **<0.001** | **1.080** | **4.871** | **<0.001** | **1.060** |
| Whether to experience medical dispute during clinical clerkship | 0.654 | 0.514 | 0.094 | 0.623 | 0.534 | 0.083 | 0.665 | 0.507 | 0.1 |

The effect size of variance analysis and t test was expressed by η²and Cohen's d separately.
